# Recognition Overdue: Military Health Records and Mortality of Norwegian Opération des Nations Unies au Congo Veterans 1960-1964

**DOI:** 10.1093/milmed/usaf387

**Published:** 2025-07-27

**Authors:** Hye Jung Choi, Elin Anita Fadum, Lene Ekhaugen, Torunn Laugen Haaland, Leif Aage Strand, Kristine Vejrup

**Affiliations:** Norwegian Armed Forces Joint Medical Services, Institute of Military Epidemiology, Sessvollmoen 2058, Norway; Department of Community Medicine and Global Health, University of Oslo, Oslo 0450, Norway; Norwegian Armed Forces Joint Medical Services, Institute of Military Epidemiology, Sessvollmoen 2058, Norway; Norwegian Institute for Defence Studies (IFS), Norwegian Defence University College, Oslo 1550, Norway; Norwegian Institute for Defence Studies (IFS), Norwegian Defence University College, Oslo 1550, Norway; Norwegian Armed Forces Joint Medical Services, Institute of Military Epidemiology, Sessvollmoen 2058, Norway; Norwegian Armed Forces Joint Medical Services, Institute of Military Epidemiology, Sessvollmoen 2058, Norway

## Abstract

**Introduction:**

As part of a Norwegian Ministry of Defence initiative, we examined the health records and mortality of veterans who served in Opération des Nations Unies au Congo (ONUC) from 1960 to 1964.

**Materials and Methods:**

We manually searched personnel folders of ONUC veterans located in the National Archives in Oslo. After transcription and quality checks of the data, we employed descriptive statistics to examine the health records assessed before and after ONUC. We linked information from 640 veterans identified in the Norwegian National Population Register to the Causes of Death Registries to analyze their mortality. Standardized Mortality Ratios (SMRs) were calculated to compare the observed number of deaths among veterans to the expected deaths among all Norwegian men. Poisson regression analysis was used to compare mortality rates across different operation-related factors and expressed as relative risk.

**Results:**

The average birth year of ONUC veterans was 1931 and the average deployment year was 1962, with deployment typically lasting 6 months. Most veterans served in the Air Force (59.4%). Their health profiles remained so following the operation and over the years. The SMR for all-cause mortality was 0.83 (95% CI: 0.75-0.90), indicating lower mortality than the general male population. There was no elevated SMR for external causes (SMR: 0.78, 95% CI: 0.50-1.15). We did not find any operation-related factors that were associated with mortality.

**Conclusion:**

To the best of our knowledge, this is the first epidemiological study using data from UN operations prior to 1978. While we experienced challenges in digitizing preserved paper-based health records for Norwegian ONUC veterans, we found that they generally had good health and lower mortality, suggesting a “healthy soldier effect.” Systematic follow-up for veterans is needed in order to preserve health data for future research and veteran welfare.

## INTRODUCTION

Opération des Nations Unies au Congo (ONUC) was established in July 1960, in response to the conflicts that erupted after Congo’s decolonization from Belgium. Following the adoption of UN Security Council Resolutions 143 and 145, Belgium was required to withdraw its troops from Congo, while the UN committed to assist the Congolese government in restoring law and order.^1^^,^^2^ Between 1960 and 1964, over 70,000 soldiers from 38 countries took part in the UN mission.^3^

Approximately 1,200 Norwegian military personnel served in ONUC.^4^ Of these, 790 were Air Force personnel and 380 were from the Army.^4^ Participation was voluntary and the Norwegians were motived primarily by a sense of adventure and economic gain.^5^ Military personnel included pilots, a movement control unit, mechanical and logistic support, and guard and patrol units.^5^

Regrettably, documentation of Norwegian ONUC veterans is scarce with limited records on personnel, their roles, experiences, and health. After homecoming, the Norwegian ONUC veterans scattered, receiving little support or recognition. To remedy this, in 2021, the Norwegian Ministry of Defence tasked the Armed Forces Joint Medical Service with reviewing archival records to identify the personnel folders in the National Archives in Oslo of unregistered veterans, transcribe the data, and include the information in the Norwegian Armed Forces Health Registry (NAFHR).

Peacekeeping service inherently carries the risk of injury or death because of war-related actions.^5–10^ Additionally, military peacekeepers may face biological, chemical, and/or psychological exposures during operations that can significantly impact their health and reduce their lifespan, even if they return home apparently healthy.^11^^,^^12^ The Norwegian Armed Forces Health Registry closely monitors mortality among Norwegian peacekeepers and have regularly published cause-specific mortality for veterans who served in Lebanon, Afghanistan, and Kosovo.^7–9^^,^^13^ This research found that military personnel generally have lower mortality compared to the general population, which is known as the ‘healthy soldier effect.’^14^^,^^15^ Nevertheless, in the first years after homecoming, the healthy-soldier-effect may level out for external causes of mortality, such as suicide or transport fatalities, and risks may even increase.^8–10^^,^^16^ Among veterans who served in Lebanon, an elevated risk of suicide was linked to conflict exposure.^9^^,^^10^ Though service in ONUC undoubtedly meant psychological stress or trauma and possible harmful physical exposures, evidence on the long-term health impacts of these veterans remains unknown.

The aim of this study was to examine the health and mortality of Norwegian veterans who served in ONUC, by analyzing information from military health assessments and the Norwegian Causes of Death Registry. To our knowledge, this is the first epidemiological study using data from UN operations prior to 1978, marking an important step in addressing a significant gap in the research literature.

## METHODS

### ONUC Veterans

The project involved collaboration between the Norwegian Armed Forces Joint Medical Service, the Norwegian Defence University College, the Norwegian Armed Forces Veterans Affairs, and the National Archives. The military archives at the National Archives were searched for information on ONUC personnel. Historical military records and health cards were dispersed across various locations, lacking a centralized system for access. The documents were digitized and uploaded to the National Archives’ digital system, facilitating the creation of a veteran cohort for further analysis.^17^

Of 1,219 ONUC veterans registered with the Armed Forces Veteran Affairs, 640 (52%) personnel folders were discovered stored in the National Archives in Oslo, Norway. These personnel folders contained either a valid personal identification number (PIN) or the member’s full name and birthdate which allowed us to link our study population to the Norwegian National Population Register and the Cause of Death Registry of Norway (**Figure S1**).

### Data Sources and Transcription

Information on the ONUC mission period and ranks was taken from the Armed Forces Veteran Affairs registrations. Health data were sourced from various military health assessment documents which we grouped into ten (10) categories based on the content: group (1) included records of military service, (2) military conscription board health examinations, (3) military health records, (4) general ability scores derived from tests of arithmetic, figural reasoning, and word similarities, (5) pre-employment medical examinations, (6) medical certificates for personnel in foreign countries, (7) Post-ONUC self-declared health status, (8) Post-ONUC medical examination in Norway, (9) military health certificates, and (10) mandatory health checks for continuously serving military personnel.

Then, the documents were grouped into 5 categories according to its purpose:

- Comprehensive military career records (1);
- Early conscript health assessment (2, 3, 4);
- Purpose-specific health examinations (5, 6);
- Health assessments post ONUC (7, 8);
- Health Check-Ups for Continuing Military Service (9, 10).

Although some documents shared similar titles and served comparable purposes, they were utilized during different time periods and under different circumstances. For instance, (3) military health records were employed in the 1940s, (9) military health certificates in the 1960s, and (10) mandatory health checks in the 1970s. Likewise, similar medical examinations were conducted (5) before employment and (6) before deployment overseas.

Each of the personnel folders enclosed various numbers of stored documents. On average, 1 folder enclosed 3 documents (SD): 1.7, with a minimum of 0 (only the folder cover was available) and a maximum of 7 documents. While some health documents included a single health record, others contained multiple records for an individual over time, particularly if the documents were used for regular health check-ups (**Table S1**).

The health documents included information measured during military health assessments, spanning from around age 18 through a military career, if the veterans continued their military careers. Height, weight, heartbeat, and systolic blood pressure (SBP) and diastolic blood pressure (DBP) were repeatedly measured. Several documents included additional information about eyesight, hearing, and any abnormalities in the heart, lungs, or abdomen.

Selected health data and information of the ONUC mission were manually transcribed. After the transcription was completed, we conducted a quality check on the transcribed data by verifying the minimum and maximum values for numeric entries and cross-referencing the scanned files if any unrealistic values were found. In cases where the handwriting was difficult to read, similar records from other health documents belonging to the same veteran were used for verification. Mortality data was retrieved from the Norwegian Causes of Death Registry. To categorize causes of death among the veterans, we applied the European short list of causes of death, which is based on different versions of the International Classification of Disease (ICD) over time.

### Statistical Analyses

Descriptive statistics of the numerical health data included average; median; minimum; and maximum values, along with the 5th and 95th percentiles. Other types of data, such as Boolean and textual information, were presented as percentages of the total number of respondents. The history of previous ­diseases was reported in several health documents; and we categorized the written diseases according to the ICD 10.

Standardized Mortality Ratios (SMR) were assessed to compare observed and expected mortality cases during the follow-up period (from January 1, 1965, to December 31, 2023, or the date of death/emigration). January 1, 1965, was selected as the start date for follow-up, as all ONUC veterans had returned to Norway by that time. Thus, one death from an accident in 1964 was not included in the analyses. The observed cases of each cause of death were compared to the expected cases of the general Norwegian male population in corresponding 5-year age bands (starting from age 20) over the same calendar years.

We also ran Poisson regression analysis to compare mortality rates across different operation-related factors, expressed as relative risk. One major operation factor was the year of deployment, as veterans who served during high-conflict periods might have experienced higher levels of stress and psychological trauma than those who served in low-conflict periods.^9^ Therefore, in Model 1, we compared veterans based on the deployment year: those deployed before and those deployed after the Katanga government’s surrender on January 14, 1963, as the conflicts between UN forces and the Katanga secessionists were particularly fierce before the surrender.^5^

Other operation-related factors were the number of places stationed (with the assumption that frequent relocations increased risk and stress), whether they were stationed in the secessionist Katanga provinces, or if they consulted a doctor during the deployment period. However, these detailed operation-related factors were only available for the 231 veterans (subpopulation) with post-ONUC self-declared health document. Therefore, these 4 factors for 231 veterans were analyzed in a sensitivity analysis by testing each variable in separate models (Models 1-4) adjusting for age at follow-up start. All 4 factors were then included in the final Model 5. All statistical analyses were conducted using Stata 17/MP.

## RESULTS

### ONUC Mission

All 640 ONUC veterans were male, and the average birth year for these veterans was 1931, with some variation observed across the rank and different regiments (**Table 1**). Each mission typically lasted for 6 months.

**Table 1.** Descriptive Statistics of 640 Opération des Nations Unies au Congo Veterans

Total of 640 veterans	*N* (%)	Mean (SD)	Median [5-95 percentiles]
Birth year	640	1930.8 (8.4)	1932 [1915-1942]
Sex (Male)	640		
Age at deployment	636	31.3 (8.4)	30 [21-47]
Year of deployment	636	1962 (1.1)	1962 [1960-1963]
Year of return	633	1962.6 (2.6)	1963 [1961-1964]
Rank			
Private	87 (13.6)	1939.6 (3.4)^a^	1940 [1932-1942]^a^
Officer and non-commissioned officer	289 (45.1)	1928.7 (6.8)^a^	1929 [1917-1939]^a^
Unknown	262 (40.1)	1930.1 (9.3)^a^	1931 [1914-1942]^a^
Types of branches			
Air Force	380 (59.4)	1930.2 (8.6)^a^	1931 [1915-1942]^a^
Ordnance	80 (12.5)	1930.3 (7.1)^a^	1932 [1917-1940]^a^
Logistics	73 (11.4)	1934.5 (6.7)^a^	1938 [1923-1941]^a^
Medical service	43 (6.7)	1926.9 (9.9)^a^	1929 [1912-1941]^a^
Headquarters Defence Command	23 (3.6)	1927.4 (4.5)^a^	1928 [1921-1934]^a^
Other	41 (6.4)	1935.6 (8.2)^a^	1940 [1920-1942]^a^

a Birth years by category.

While the Veteran Affairs database contained operational period information for the study population, we also found operational period data for 231 (36.1%) veterans—those who had both health documents (Types 7 and 8) assessed upon returning to Norway after the ONUC mission. The average deployment and return year for these 231 veterans was 6 months later (**Table 2**, left) than those recorded in the Veteran Affairs list (**Table 1**), whereas the operation duration was still typically 6 months.

**Table 2.** Detailed Information About Opération des Nations Unies au Congo (ONUC) Mission

(7) Post-ONUC self-declared health status (*n* = 231)		(8) Post-ONUC medical examination in Norway (*n* = 232)	
Year of assessment (SD)	1963.4 (0.5)	Year of examination (SD)	1963.3 (0.4)
Year of deployment (SD)	1962.7 (0.6)	Overall health status (%)	
Year of return (SD)	1963.1 (0.4)	Very good	28 (12.1)
Number of stationed sites (%)		Good	188 (81)
1	123 (53.2)	OK	2 (0.9)
2	36 (15.6)	Missing	14 (6)
3	23 (10)	Any health issues at the examination	
4 and more	28 (12.1)	Yes	26 (11.2)
Missing	21 (9.1)	- Diarrhea	5 (2.1)
Types of services (%)		- Cold, coughing	4 (1.7)
Mechanics, technicians	37 (16)	- Injuries	5 (2.2)
Logistics, movement control	26 (11.2)	- Eye and ear	3 (1.3)
Pilot, air operation	25 (10.8)	- Genital organ	3 (1.3)
Guard, patrol	22 (9.5)	- Skin disease	3 (1.3)
Administration	20 (8.7)	- Others	3 (1.3)
Military police	18 (7.8)	No	188 (81)
Operations	11 (4.8)	Missing	18 (7.7)
Infantry	5 (2.1)	Feel healthy at the examination	
Communication	5 (2.1)	Yes	189 (81.5)
Medical	3 (1.3)	No	37 (16)
Others	27 (11.6)	Missing	6 (2.6)
Missing	32 (13.8)	Hemoglobin level	190 (81.9)
		Mean^%^(SD)	106 (7.8)
		Median [5-95 percentiles]	105 [95-120]
		Erythrocyte Sedimentation Rate	93 (40)
		Mean^m^(SD)	5.5 (5.9)
		Median [5-95 percentiles]	4 [1-20]

Abbreviations:^%^, expressed as a percentage; ^m^, expressed as mm.

Approximately 53% of the veterans who held the self-declaration document were stationed in a single location, most commonly Leopoldville (now Kinshasa) (**Table 2**, left). Their health status appeared to be generally good (93.1%), and 81.5% reported feeling healthy during the medical examination upon their return (**Table 2**, right). Two clinical test results were available—hemoglobin levels and erythrocyte sedimentation rate; however, these tests involved only a small number of participants, and no information was found regarding the testing methodologies, measurement instruments, or procedural protocols (**Table 2**, right).

### Health Records

Key health metrics were measured across various health documents, with examinations ranging from the 1940s to the 1980s. Height and heart rate remained relatively consistent, while weight, SBP, and DBP showed increases over time, reflecting lifestyle and age-related changes (**Table 3**). In addition to this, 128 had a stanine score for general ability recorded in their military records. The stanine scale ranges from 1 to 9 and is comparable to IQ; a stanine of 5 corresponds to an IQ of 100 on the WAIS (Wechsler Adult Intelligence Scale) scale, with each stanine unit representing ±7.5 IQ points.^18^ The mean stanine score was 6.4 (SD: 1.6), with a median of 6 [5-95% percentile: 4-9], indicating that these 128 veterans generally exhibited above-average cognitive ability.

**Table 3.** Health Records of the ONUC Veterans

	(2) Military medical examinations at the conscription board		(3) Military health records		(5) Pre-employment medical examinations		(6) Medical certificate for personnel in foreign countries		(8) Post-ONUC medical examination in Norway^a^		(9) Military health certificates		(10) Mandatory health checks for continuously serving military personnel	
	Mean	Median	Mean	Median	Mean	Median	Mean	Median	Mean	Median	Mean	Median	Mean	Median
Year of examination	1953.9(6)	1954[1946-61]	1945.6(0.8)	1946[1945-46]	1962.6(3.2)	1962[1958-68]	1961.3(4.1)	1963[1952-65]	1963.3(0.4)	1963[1963-64]	1961.2(3.7)	1962[1954-67]	1976(4.8)	1976[1969-83]
Height (cm)	178.1(6.4)	178[168-169]	176.5(6)	178[165-187]	–	–	178.6(5.7)	179[170-188]	–	–	180.1(6.4)	180[170-192]	178(5.5)	178[169-188]
Weight (kg)	70.2(8)	70[58.2-85]	68.5(7.7)	67[58.5-82]	–	–	74.4(8.2)	73[63-89]	–	–	76.2(8.2)	75[54-90]	78.5(9.6)	78[63-96]
SBP (mm Hg)	128.6(12.7)	130[110-150]	125.1(9.1)	125[110-140]	128.8(14.6)	125[110-155]	127.6(12.1)	125[110-150]	125.6(9.7)	127.5[110-140]	128.6(11.9)	130[110-150]	137.6(18.2)	135[115-170]
DBP (mm Hg)	78.5(8.5)	80[65-90]	75.2(7)	75[65-85]	79.8(9.3)	80[65-95]	79.2(8.7)	80[70-95]	79.4(7.4)	80[60-90]	79.9(7.6)	80[70-90]	87.1(10.9)	85[70-105]
Heart rate (bpm)	73.8(10.2)	72[60-90]	–	–	–	–	73.3(8.7)	72[60-88]	82(14.1)	84[60-112]	–	–	70.9(9.4)	72[58-88]

Mean (SD) and median [5-95 percentiles].

a The Post-ONUC medical examination in Norway form did not include health indicators of SBP, DBP, and heart rate. However, notes on the form contained this information for 32 out of 232 individuals, which is summarized here.

Abbreviations: DBP, diastolic blood pressure; ONUC, Opération des Nations Unies au Congo; SBP, systolic blood pressure.

Five health documents had a question about the types of diseases veterans had before their examinations, and it was common for them to report multiple conditions (**Table S2**). Among those who reported their previous diseases during the conscription board examination, respiratory diseases were the most commonly reported (34%), followed by injuries (28%). As the examination for the “Medical Certificate for Personnel in Foreign Countries” was primarily conducted before veterans were deployed abroad, a significant number reported that they had undergone an appendectomy thus they had scars on their bodies. This accounted for a large portion of the reported cases of digestive diseases (33%) and notes irrelevant to diseases (30%). In 4 health documents, common childhood diseases (ICD-10: A00-A99) were frequently reported, except in the post-ONUC examination. In the post-ONUC self-declared health, parasitic diseases (ICD-10: B00-B99), particularly mycoses, were the most prevalent, accounting for 19% of cases where veterans consulted a doctor while in Congo, followed by digestive diseases (16%), primarily diarrhea.

### Mortality

From the beginning of 1965 to the end of 2023, 480 (75.1%) of the veterans that we reviewed had died, 148 (23.2%) were alive, and 11 (1.7%) had emigrated thus their vital status is unknown. The mean age at death was 76.1 years, with a SD of 12 years. Cardiovascular disease (CVD) was most common causes of death, followed by cancer (**Table 4**). Of the 21 mortality cases due to mental and behavioral disorders, only 1 was attributed to chronic alcohol abuse and alcohol-related psychosis. Among the 25 deaths from external causes, 6 were suicides and 2 were poisoning accidents.

**Table 4.** Standard Mortality Ratios for All-Cause and Cause-Specific Mortality Among the Opération des Nations Unies au Congo Veterans (Person-Years = 28,187)

	Observed	Expected	SMR (95% CI)
**All-cause mortality**	480	580.64	0.83 (0.75-0.90)^a^
Causes not registered	21	5.69	3.69 (2.28-5.64)^a^
External causes (accidents, poisonings, suicides)	25	32.17	0.78 (0.50-1.15)
Disease related	434	542.77	0.80 (0.73-0.88)^a^
Infectious and parasitic diseases	4	9.72	0.41 (0.11-1.05)
Cancers	150	164.5	0.91 (0.77-1.07)
Endocrine, nutritional, and metabolic diseases	9	11.79	0.76 (0.35-1.45)
Mental and behavioral disorders	21	20.21	1.04 (0.64-1.59)
Nervous system and sensory organs	19	19.26	0.99 (0.59-1.54)
CVD	154	209.81	0.73 (0.62-0.86)^a^
Respiratory organs	41	59.29	0.69 (0.50-0.94)^a^
Digestive organs	7	16.12	0.43 (0.17-0.89)^a^
Skin and subcutaneous tissue	1	0.82	1.22 (0.03-6.80)
Skeleton, muscles, and connective tissue	3	2.52	1.19 (0.25-3.48)
Urinary and genitals	12	11.06	1.09 (0.56-1.90)
Congenital malformation and chromosomal anomalies	1	0.69	1.45 (0.04-8.06)
Symptoms and undetermined conditions	10	18.52	0.54 (0.26-0.99)^a^
COVID-19	2	2.73	0.73 (0.09-2.65)

a *P*-value < .05.

Abbreviations: CI, confidence interval; CVD, Cardiovascular disease.

The SMR for all-cause mortality was 0.83 (95% confidence interval: 0.75-0.90), indicating lower mortality than expected. Disease-related mortality had an SMR of 0.79 (0.72-0.87). In contrast, deaths without registered causes were significantly higher than in the general population (SMR: 3.69, 95% CI: 2.28-5.64). In terms of cause-specific mortality, deaths from CVD were significantly lower than expected (0.73, 0.62-0.86). Similarly, mortality from respiratory organ diseases was 31% lower (0.69, 0.50-0.94), and mortality from digestive organ diseases was 57% lower (0.43, 0.17-0.89) compared to the expected cases.

We attempted to analyze SMRs based on different follow-up periods (1 year, 5 years, and 10 years), however, only a small number of deaths—15 (3%)—occurred between 1965 and 1975.

We found no difference in the relative risk of death between veterans deployed during the 2 different time periods, before/after 1963. However, older age was associated with higher mortality (**Table S3**). When analyzing detailed operation-related factors from the post-ONUC document of those 231 who provided them, none were associated with mortality, except for age (**Table S4**).

## DISCUSSION

### Principal Findings

In collaboration, the Norwegian Armed Forces Joint Medical Service, the Norwegian Defence University College, the Norwegian Armed Forces Veterans Affairs, and the National Archives, we have identified health records for 640 ONUC veterans. We then transcribed and analyzed both their operation records and health data measured from military health assessments over the years. The veterans generally maintained good physical health and had lower all-cause mortality compared to the general male population. We did not find statistically significant associations between operation-related factors and mortality. However, most analyses were conducted on a sub-population with post-ONUC self-declared health document.

### Comparison With Previous Studies

We observed a ‘healthy soldier effect,’ consistent with findings from previous studies on Norwegian and Nordic veterans.^7^^,^^8^^,^^13^^,^^16^ Among 21,609 Norwegian male military peacekeepers deployed to Lebanon between 1978 and 1998, the SMRs for all-cause mortality and disease-related mortality were 0.85 and 0.76, respectively,^7^ closely aligning with our findings. Moreover, Norwegian veterans deployed to Afghanistan between 2001 and 2019 had an SMR of 0.56 for all-cause mortality after discharge from peacekeeping service,^8^ while peacekeepers in Kosovo between 1999 and 2016 had an SMR of 0.62.^13^ These observed lower mortality among military personnel was often described as a ‘healthy soldier effect,’ resulting from military selection and the demand to maintain physical fitness for those continuing their military careers.^6–8^^,^^13^^,^^14^

Yet, it should be noted that lower mortality does not fully capture the holistic health of veterans, which encompasses mental health conditions, emotional, and social well-being. Indeed, mental health problems among veterans of foreign conflicts have been frequently reported and studied.^11^^,^^19^^,^^20^ In a 2020 survey conducted for Norwegian veterans deployed to Afghanistan from 2001 to 2020, 10.4% (*n* = 635) of veteran participants reported having at least one mental health crisis at the time of the survey. The survey also showed that mental health issues were more prevalent among those who left the Armed Forces after deployment (14.9%) compared to those who continued their military career (8.2%), with this difference being statistically significant.^19^ In contrast, a 2012 survey conducted by Statistics of Norway on the living conditions for veterans who served in international operations from 1978 to 2012 found that, among 1,851 veterans interviewed, their physical and mental health was as good as their reference group, which had the same gender and age distribution.^20^

Although there are no specific surveys or studies on the mental health of ONUC veterans, a 2016 survey for veterans served in the United Nations Interim Force in Lebanon (UNIFIL) in 1978-1998 included 18 ONUC veterans who also served in UNIFIL. Although the ONUC sample size was small, the survey found that only 1 (5.6%) of the 18 veterans reported experiencing anxiety or depression in the past week, 2 (11.1%) had attempted suicide ever, and 4 (22.2%) reported harmful alcohol use in the past year.^17^ Overall, the survey showed that UNIFIL veterans were satisfied with their lives and health, and did not report suffering from severe illnesses or injuries, similar to previous studies on Norwegian peacekeepers.^21^

Previous studies among U.S. veterans often found an elevated risk of external-cause mortality, such as suicide and accidents, after discharge compared to the general population.^22^^,^^23^ This was also found among Norwegian UNIFIL veterans exposed to high-conflict environments in the short period after discharge.^9^ Increased external-cause mortality was often attributed to traumatic or violent events during deployment,^9^ and mental health disorders^24^ among veterans. However, we found neither higher external-cause mortality among veterans nor any operation-related factors associated with mortality. In our study, there were 9 deaths due to chronic alcohol abuse and alcohol-related psychosis, poisoning, and suicides, which occurred a median of 21 years after their return. Of these 9 cases, only 2 suicides occurred during the first 10-year follow-up period. This could indicate that the veterans included in this study did not experience severe mental or behavioral health problems that led to death. However, due to the lack of data on mental health and lifestyle from systematic follow-up for all ONUC veterans these assumptions remain inconclusive.

### Strengths and Limitations

To our knowledge, this is the first epidemiological study using data from UN operations prior to 1978, filling a significant gap in the research literature. The scattered and scarce records of ONUC veterans have made it difficult to conduct follow-ups and to investigate their health. Even the total number of Norwegian ONUC veterans is uncertain, with 1,219 registered by the Veteran Affairs and 1,173 found in a previous study from 1972.^4^ This discrepancy resulted from a lack of, or incomplete, overview of veterans records in the past, which has been a major challenge in Norwegian veteran studies.^25^ For this review, we located and digitized the health and operation records of presumptively 50% of the ONUC veterans who took part in Norway’s first UN mission. This newly digitized data will be registered in the NAFHR and can serve as a valuable resource for future research.

Furthermore, we had records for an extensive follow-up period for mortality, spanning 58 years from 1965 to 2023. By linking participants with their unique PINs to the national death registry, we were able to capture long-term trends and mortality outcomes. Quality checks were conducted during the transcribing and digitizing process, ensuring the accuracy and integrity of the data, minimizing potential errors, and enhancing the reliability of the study findings.

Several limitations remain and should be addressed, including that we were unable to include all 1,219 veterans from the ONUC mission, identifying only 640 (52.5%) for this study. Despite our efforts, the absence of valid PINs and incomplete personal information hindered our ability to link the records of those we could not find to the registers. When comparing the veterans included in our study with those not included, the latter group was deployed to Congo 1 year earlier than those in our study population. In addition, only one veteran deployed to Congo in 1961 possessed 2 post-ONUC documents, and there were no records from these documents dated before 1962. This indicates that the archival process and health measurements for the ONUC mission have been implemented midway through the operation, rather than at the outset. Alternatively, it is also possible that the veterans we could not identify, or those with minimal records, may be located in other archives.

Additionally, if clinical, psychological, pharmaceutical, or other health related data had been available, we could have analyzed diverse health impacts on the veterans, including exposure to health hazards or traumatic experiences during their missions. However, due to the limited availability of clinical data for only a select group, the lack of conducted test information, and the absence of systematic follow-up, an in-depth analysis of their health outcomes was unfortunately not possible.

The number of records and documents available for each veteran varied by their branches of service and on where their personnel folders were stored. Veterans from the Air Force typically had the most extensive records and documents, as they tended to have longer military careers. Although this seems plausible given that members of the Air Force made up the majority of ONUC veterans. Veterans who left military service after ONUC and those with fewer records from other regiments may be underrepresented in this study.

Although SMRs for several types of deaths suggest that the veterans that we looked at were relatively healthy, we cannot rule out the potential impact of those not included in this study. The relatively high proportion of deaths with causes not reported (21 observed vs. 5.69 expected) have underestimated the SMRs for specific causes. The SMR for all causes, including those with unknown causes (SMR = 0.83), decreases to 0.80 (0.73-0.88) when excluding these unknown causes, indicating the ‘healthy soldier effect’ is overestimated by 3%. Of the 21 deaths with unregistered causes, 16 lacked accompanying death certificates, and most cases of this kind occurred abroad.^26^^,^^27^

## CONCLUSION

Health records of the Norwegian ONUC veterans were poorly preserved. Records were scattered, the health documents in each personnel folder varied extensively, and documents were scarce. A meticulous effort aiming to identify Norwegian ONUC veterans resulted in the identification of 640 out of approximately 1,200 veterans. The records show that the veterans included in this study were, in general, in good health. The veterans also had lower mortality compared to the general male population. These findings indicate a “healthy soldier effect” among a Norwegian veteran group from before 1978.

Upon returning home, the ONUC veterans received little follow-up from the Armed Forces, or recognition from the Norwegian society. However, the potential harmful effects on soldiers’ health from participating in peacekeeping operations were largely unknown until the late 1980s, when Norway began to examine the health of Norwegian participants in the UNIFIL operation in Southern Lebanon.^28^ As a result, some veterans felt forgotten, as their service remained unrecognized. In a 2022 documentary, a ONUC veteran expressed his disappointment, exclaiming that “they [the Norwegian Armed Forces] didn’t even know which of us were there.”^29^ Therefore, it is crucial to maintain systematic record-keeping and follow-up with veterans after their service. This acknowledges their contributions and can also contribute to preserve health data that can inform future research and enhance veteran welfare.

## Supplementary Material

usaf387_Supplementary_Data

## Data Availability

The datasets generated and/or analyzed during the current study are not publicly available but are available from the Norwegian Armed Forces Health Registry with necessary approval.

## INSTITUTIONAL CLEARANCE

The institutional clearance has been approved.
