# Supplementary material for: Recognition Overdue: Military Health Records and Mortality of Norwegian Opération des Nations Unies au Congo Veterans 1960-1964
Source: Mil Med. 2025 Jul 27;191(1-2):e396–403. doi: 10.1093/milmed/usaf387 (PMC12826853; doi:10.1093/milmed/usaf387)
Supplement: usaf387_Supplementary_Data [file usaf387_supplementary_data.zip › Supplementary materials_Norwegian ONUC veterans_Clean.docx]

[Figure S1. The flow chart of ONUC veterans included in the study population]


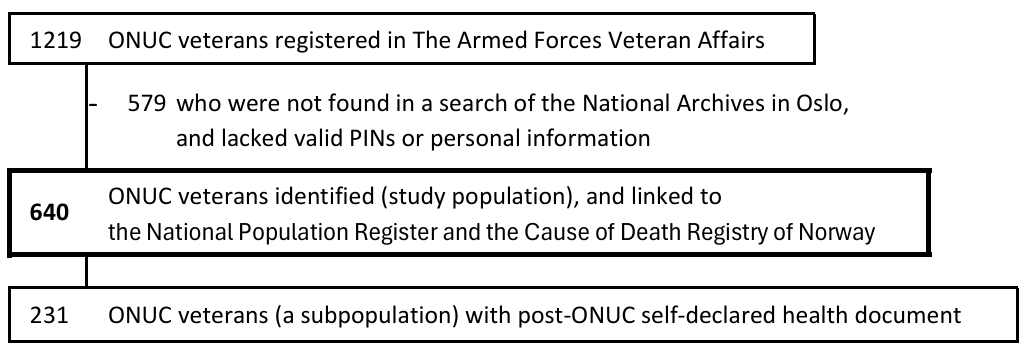


[Table S1. Types of health documents]

| Type | Description | 640 veterans | | | 4289 records | |
| --- | --- | --- | --- | --- | --- | --- |
|  |  | n | | % | n | % |
| (1) | Military service records | 568 | 88.8 | | 568 | 13.2 |
| (2) | Military conscription board health examinations | 461 | 72 | | 463 | 10.8 |
| (3) | Military health records | 33 | 5.2 | | 33 | 0.8 |
| (4) | General ability tests | 39 | 6.1 | | 39 | 0.9 |
| (5) | Pre-employment medical examinations | 135 | 21.1 | | 222 | 5.2 |
| (6) | Medical certificate for personnel in foreign countries | 294 | 45.9 | | 350 | 8.2 |
| (7) | Post-ONUC self-declared health status | 231 | 36.1 | | 231 | 5.4 |
| (8) | Post-ONUC medical examination in Norway | 232 | 36.3 | | 233 | 5.4 |
| (9) | Military health certificates | 168 | 26.3 | | 276 | 6.4 |
| (10) | Mandatory health checks for continuously serving military personnel | 202 | 31.6 | | 1874 | 43.7 |

[Table S2. Descriptive statistics on reported history of disease among the ONUC veterans]

|  | (2) Military medical examinations at the conscription board | (3) Military health records | (6) Medical certificate for personnel in foreign countries | (7) Post-ONUC self-declared health status^§^ | (9) Military health certificates |
| --- | --- | --- | --- | --- | --- |
| Mean year of examination (SD) | 1953.9 (6) | 1945.6 (0.8) | 1961.3 (4.1) | 1963.5 (0.7) | 1961.2 (3.7) |
| Mean age at measurement (SD) | 20.2 (4.5) | 22.4 (4.2) | 29.6 (8) | 29.7 (7.4) | 28.4 (6.5) |
| Total numbers of veteran reported (%^‡^) | 267 (62^‡^) | 15 (45^‡^) | 122 (35^‡^) | 124 (55^‡^) | 88 (33^‡^) |
| Certain infectious and parasitic diseases (A00-B99) | 44 (16) | 8 (53) | 1 (0.8) | 24 (19) | 19 (12) |
| Diseases of the eye and adnexa (H00-H59) | 25 (9) |  | 10 (8) | 1 (1) | 3 (2) |
| Diseases of the ear and mastoid process(H60-H95) | 21 (8) |  | 5 (4) | 10 (8) | 6 (4) |
| Diseases of the respiratory system (J00-J99) | 91 (34) |  | 4 (3) | 13 (10) | 11 (7) |
| Diseases of the digestive system (K00-K93) | 32 (12) | 3 (20) | 40 (33) | 20 (16) | 18 (11) |
| Diseases of the skin and subcutaneous tissue (L00-L99) | 4 (1) |  | 1 (0.8) | 14 (11) | 1 (1) |
| Diseases of the musculoskeletal system and connective tissue (M00-M99) | 19 (7) |  | 5 (4) | 1 (1) | 10 (6) |
| Diseases of the genitourinary system (N00-N99) | 6 (2) |  | 1 (0.8) | 6 (5) | 3 (2) |
| Injury, poisoning and certain other consequences of external causes (S00-T98) | 74 (28) | 3 (20) | 12 (19) | 14 (11) | 17 (10) |
| Symptoms, signs and abnormal clinical and laboratory findings,  not elsewhere classified (R00-R99) | 13 (5) | 2 (13) | 4 (3) | 4 (3) | 3 (2) |
| Other diseases not included above (CVDs, obesity, etc.) | 5 (1) |  | 6 (5) | 22 (18) | 15 (9) |
| Notes irrelevant to disease | 20 (7) | 1 (7) | 37 (30) | 7 (5) | 2 (1) |

^§^The Post-ONUC self-declared health: asked veterans to report any diseases for which they saw a doctor during ONUC in Congo.

The (%‡) indicates the percentage of veterans reporting a disease among those who answered (yes, no) to a question about their history of diseases in each corresponding form.

[Table S3. Relative risks (95%CI) for all-cause mortality among the ONUC veterans (480 deaths), expressed as rate ratios]

|  | Model 1 | Model 2 |
| --- | --- | --- |
| Deployment year |  |  |
| 1960-1962 (n=368) | ref | ref |
| 1963-1964 (n=267) | 0.94 (0.79-1.13) | 0.99 (0.82-1.20) |
| Missing (n=4) | 0.89 (0.29-2.78) | 0.95 (0.30-2.99) |
|  |  |  |
| Age in 1965 |  |  |
| 20-29 (n=254) | - | ref |
| 30-39 (n=234) |  | 1.10 (0.88-1.38) |
| 40-61 (n=151) | - | 1.43 (1.12-1.82)^*^ |

^*^P-value < 0.05

[Table S4. Relative risks (95%CI) for all-cause mortality among 231 ONUC veterans (159 deaths), with post-ONUC document (subpopulation) expressed as rate ratios]

|  | Model 1 | Model 2 | Model 3 | Model 4 | Model 5 |
| --- | --- | --- | --- | --- | --- |
| Deployment year |  |  |  |  |  |
| 1960-1962 (n=67) | ref |  |  |  | ref |
| 1963-1964 (n=163) | 1.15 (0.81-1.63) |  |  |  | 1.16 (0.81-1.65) |
| Missing (n=1) | 1.47 (0.20-10.8) |  |  |  | 1.29 (0.16-10.1) |
|  |  |  |  |  |  |
| Katanga region |  |  |  |  |  |
| Yes (n=134) |  | ref |  |  | ref |
| No (n=76) |  | 0.89 (0.63-1.26) |  |  | 0.98 (0.65-1.46) |
| Missing (n=21) |  | 1.02 (0.59-1.73) |  |  | 1.15 (0.62-2.12) |
|  |  |  |  |  |  |
| Number of sites |  |  |  |  |  |
| 1 (n=123) |  |  | ref |  | ref |
| 2 and more (n=87) |  |  | 1.19 (0.85-1.65) |  | 1.18 (0.80-1.74) |
| Missing (n=21) |  |  | 1.14 (0.67-1.96) |  | omitted |
|  |  |  |  |  |  |
| Consulted a doctor |  |  |  |  |  |
| No (n=102) |  |  |  | ref | ref |
| Yes (n=124) |  |  |  | 0.92 (0.67-1.27) | 0.89 (0.64-1.24) |
| Missing (n=5) |  |  |  | 0.88 (0.32-2.41) | 0.86 (0.30-2.42) |
|  |  |  |  |  |  |
| Age in 1965 |  |  |  |  |  |
| 20-29 (n=118) | ref | ref | ref | ref | ref |
| 30-39 (n=80) | 2.05 (1.44-2.91)^*^ | 2.06 (1.45-2.94)^*^ | 2.04 (1.43-2.90)^*^ | 2.07 (1.46-2.95)^*^ | 2.08 (1.46-2.98)^*^ |
| 40-61 (n=33) | 3.08 (2.00-4.76)^*^ | 3.06 (1.97-4.75)^*^ | 3.05 (1.98-4.69)^*^ | 2.96 (1.92-4.54)^*^ | 3.15 (2.01-4.93)^*^ |

^*^P-value < 0.05
